# Efficacy and cost savings with the use of a minimal sedation / anxiolysis protocol for intra-articular corticosteroid injections in children with juvenile idiopathic arthritis: a retrospective review of prospectively collected data

**DOI:** 10.1186/s12969-019-0312-y

**Published:** 2019-03-20

**Authors:** Rotem Elitsur, April Hollenbeck, Laura Tasan, Kathryn S. Torok, Elaine Cassidy, Brian Blasiole, Erika Parsons, Chelsea Acock, Joseph Angelelli, Isabela-Cajiao Angelelli

**Affiliations:** 1 Department of Pediatrics - Division of Pediatric Emergency Medicine, UPMC Children’s Hospital of Pittsburgh, 4401 Penn Avenue Pittsburgh, Pittsburgh, PA 15224 USA; 2 Department of Pediatric Anesthesiology, UPMC Children’s Hospital of Pittsburgh, 4401 Penn Avenue Pittsburgh, Pittsburgh, PA 15224 USA; 3 Department of Pediatrics - Division of Pediatric Rheumatology, UPMC Children’s Hospital of Pittsburgh, 4401 Penn Avenue Pittsburgh, Pittsburgh, PA 15224 USA; 4 Department of Pediatric Radiology, UPMC Children’s Hospital of Pittsburgh, 4401 Penn Avenue Pittsburgh, Pittsburgh, PA 15224 USA; 5 UPMC Center for High Value Health Care, UPMC Health Plan, 600 Grant St, Pittsburgh, PA 15219 USA

**Keywords:** Anxiolysis, Nitrous oxide, Fentanyl, Child life specialist, Intra-articular corticosteroid injection, Joint injection, Child, Cost savings, High-value care

## Abstract

**Background:**

Intra-articular corticosteroid injections (IACI) are frequently used in the treatment of juvenile idiopathic arthritis. There is a paucity of evidence-based research describing methods of pain and anxiety control for this procedure. IACI were mostly performed under general anesthesia for children younger than 13 years old in our institution as of 2014. We started to integrate sedation services more commonly in our institution with the minimal sedation/anxiolysis (MSA) protocol outlined as an alternative to general anesthesia for IACI in 2015. The purpose of this study was to evaluate the effectiveness and cost savings of a minimal sedation protocol for intra-articular corticosteroid injections in juvenile idiopathic arthritis patients after instituting this protocol at our institution.

**Methods:**

The MSA protocol included nitrous oxide, intranasal fentanyl, a topical numbing agent, acetaminophen, ibuprofen, ondansetron and child life intervention. A retrospective review of prospectively collected data was performed on a total of 80 consecutive patients with juvenile idiopathic arthritis who underwent joint injections using the protocol.

**Results:**

The procedure was successfully completed in greater than 95% of the patients. The median pain score (measured on a verbal numeric scale of 0–10) reported by the patient was 1 (IQR 0–2.5), by the parent 1 (IQR 0–2), by the rheumatologist 1 (IQR 0–1), and by the sedationist 1 (IQR 0–1). Degree of motion during the procedure was reported by the rheumatologist and the sedationist as none in 68% of the patients, mild in 36% and moderate in 6%. Patient, parent, rheumatologist and sedationist rated satisfaction as very high in the vast majority (94%). Emesis was reported in only 2 (2.5%) patients, no significant adverse events were reported, and no patients progressed to a deeper level of sedation than intended. Financial analysis revealed a 33% cost reduction compared with the use of general anesthesia in the operating room.

**Conclusions:**

A minimal sedation/anxiolysis protocol (including nitrous oxide, intranasal fentanyl, a topical numbing agent, acetaminophen, ibuprofen, ondansetron and child life intervention), provides safe and effective analgesia for intra-articular corticosteroid injection in a subset of patients with juvenile idiopathic arthritis and offers a lower cost alternative to general anesthesia.

## Background

High-value care has emerged as a new ethos for practicing medicine, with a greater focus on minimizing waste, containing costs, reducing medical errors, strengthening the use quality metrics and improving population health. It emphasizes non-maleficence, or doing no harm to patients, by reducing overutilization of tests and unnecessary care [1]. One area of opportunity is to address the Sedation Service’s potential to help reduce patient pain and anxiety in a safe and effective manner outside of the operating room (OR).

Intra-articular corticosteroid injection (IACI) for rheumatology patients with juvenile idiopathic arthritis (JIA) is an example of a minimal sedation/anxiolysis (MSA) sensitive procedure. JIA is the most common rheumatologic disease in children. Left untreated it can lead to joint damage impacting a child’s quality of life and eventually leading to disability [2]. ICAIs are frequently used in the treatment of JIA to help to reduce local inflammation, which relieves pain and helps to preserve joint function. These disease-modifying injections have the unfortunate consequence of inducing a great deal of anxiety and pain in the pediatric population [3]. Currently, there is no accepted standard of care in providing general anesthesia, anxiolytics or sedation for these patients [4, 5]. At our institution, historically the procedure was performed in the OR under general anesthesia for younger patients (generally 12 or younger) or awake in the rheumatology office with no anxiolytic for those 13 and older. In addition to younger age, those with more than 2 joints to inject and with small joints to be injected, such as interphalangeal joints or temporomandibular joints, were typically assigned to general anesthesia.

Painful procedures are a major source of distress in children. Once pain has been under treated, it becomes harder to treat, even with the same noxious stimulus [6], furthermore complicating an in office IACI. Building onto this anticipation of pain, specifically in the JIA population, is development of “needle phobia” derived from their frequent phlebotomy draws for medication monitoring or due to delivery of immunosuppressive medication (subcutaneous injections or intravenous route). Therefore, the combination of learned pain (having an IACI procedure prior in the office without any sedation) and needle phobia, was setting up many JIA patients as ‘poor cooperation’ candidates for in office IACI, leading ultimately to the decision of general anesthesia for repeat joint injections. Therefore, a large portion of JIA patients underwent general anesthesia in the OR for the relatively short IACI procedure, adding potential respiratory and cardiovascular risk, and prolonging this intervention to several hours, with total time in the hospital for the procedure frequently being greater than 5 h when including check-in process and recovery time. As pediatric rheumatologists are sparse, several of patients require additional travel time further adding to the already long day/displeasure of undergoing procedures.

There is a paucity of research describing methods of pain and anxiety control for this procedure. Three international studies (outside of the USA) describe the use of nitrous oxide (N_2_O) [7–9]. These studies concluded that N_2_O can be used to safely and effectively provide analgesia for IACI in children. However, there is still a significant number of patients who experience significant pain during the procedure [9]. Theoretically, the addition of intranasal (IN) administration of fentanyl, as well as oral acetaminophen and ibuprofen is attractive, as it would improve pain control, and these methods were incorporated into our MSA protocol. Previous studies have reported on the effectiveness of IN fentanyl/N_2_O combination for more painful procedures, like forearm fracture reductions [10]. As the combination of IN fentanyl/N_2_O is associated with an increased incidence of emesis, the use of ondansetron has been recommended [10], and therefore, ondansetron is also included in our MSA protocol. The addition of distraction techniques through active participation of a medical clown during IACI with N_2_O has shown to further decrease pain and stress [8], prompting the incorporation of Child Life Services for distracting techniques for our MSA protocol.

**Fig. 1:**
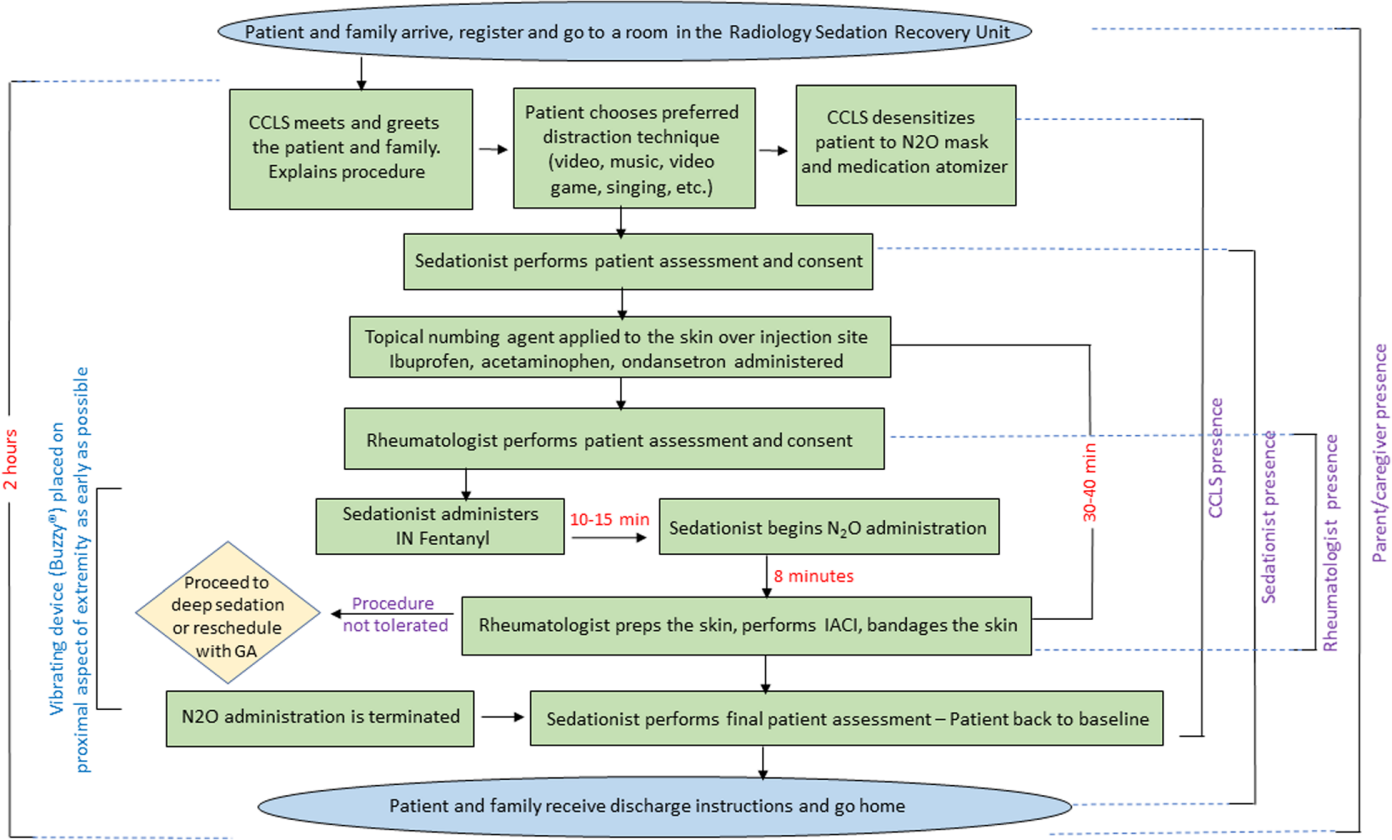
MSA protocol and IACI process flow diagram

The Sedation Service at our institution embarked on the utilization of modified MSA protocol in 2015, which incorporated the above techniques (see Fig. 1), to assist the Pediatric Rheumatology Division in the care of the pediatric population with JIA during IACI without the need of general anesthesia. This is particularly relevant because parents, in general, prefer to avoid the inherently increased risks of general anesthesia for IACI [3].

In this study, we report our experience with the use of this MSA protocol in 80 consecutive JIA patients who required IACI. The protocol developed includes the use of N_2_O along with topical numbing cream, IN fentanyl, acetaminophen, ibuprofen, ondansetron, preparation and distraction techniques by a certified child life specialist (CCLS) (Fig. 1). We evaluate the implications of this protocol by assessing safety, patient satisfaction and cost reduction incurred when performing the procedure outside of the operating room.

## Patients and methods

This retrospective chart review of prospectively collected data included children 18 years of age and younger with a diagnosis of JIA who required one or more IACIs and presented to the Sedation Service at our center which is a 415 bed, tertiary medical center and the only hospital in our region dedicated solely to the care of infants, children and young adults. The Pediatric Sedation Service takes care of over 2000 patients every year. Most of these patients are taken care of within the different units in the Pediatric Radiology Department.

Institutional Review Board (IRB) approval was obtained prior to conducting the study. The requirement to obtain written informed consent was waived by the IRB as this chart review involved minimal risk to subjects and could not be carried out practically without the waiver. The study included 80 consecutive patients who presented from December of 2015 to September of 2017. No patient charts were excluded from the review. A list of all the patients that had an IACI under the MSA protocol were identified from the Sedation Service and Pediatric Rheumatology Service patient logs and these electronic medical records were reviewed.

Data collected prospectively included: patient’s age, number of joints injected, degree of pain, amount of motion during the procedure and satisfaction with the procedure. A pain score was measured using a discrete verbal numeric rating (VNR) scale from 0 to 10 (where 0 represents no pain and 10 represents severe pain) as perceived by the patient, parent, rheumatologist and sedationist. When children showed difficulty comprehending the scale, the pain score was reported as “unable to evaluate” and was excluded from the analysis. In addition to determining the median and IQR of the pain score, the pain score was categorized into mild-moderate-severe, as described by Tsze et al. [10], with pain in the range of 0 to 3 considered as mild, the range of 4 to 6 as moderate and the range of 7 to 10 as severe. A minor modification was made to score categorization, in which a ‘none’ category was added based on the floor effect demonstrated in the data, with most patients rating pain as “0”, and mild then contained only scores of 1 to 3. Amount of motion during the procedure was measured independently as perceived by the rheumatologist and the sedationist using a four-grade modified Likert scale (none, mild, moderate, severe). Satisfaction with the procedure was measured as reported independently by the patient, parent, rheumatologist and sedationist using a four-grade modified Likert scale (very satisfied, satisfied, somewhat satisfied, not satisfied). The respondents answered the questions independent of one another. Adverse event data were collected as well, including oxygen desaturation, change greater than 20% in heart rate, emesis, agitation, and change in the intended level of sedation. Data analysis of these clinical and patient-reported outcomes included descriptive statistics, with median and IQRs reported as the data were not normally distributed.

To assess for the degree of change in general anesthesia utilization for IACIs, the number of patients that were referred to the anesthesiology service during the time frame of the study was obtained from the operating room scheduling records of the Pediatric Rheumatology Division at our institution and compared to the baseline percentage utilization of that service.

Cost analysis was performed by using our institution’s Cost Management System data as of October of 2017. Total expense to hospital was calculated by determining the following: direct supply expense, direct drug expense, salary expenses, unit operating expenses, utilities and maintenance expenses, and indirect expenses. Total expense to hospital was calculated for 15 patients who underwent IACI under general anesthesia in the Procedure Center (equivalent to an OR setting) during the same time period as the patients who had IACI with our MSA protocol performed in the Radiology Sedation Recovery Unit. As the administrative process for billing of our procedure with MSA was not in place yet, this cost was obtained by extrapolating the total expense to the hospital of performing auditory brain stem response testing (ABR) for 15 patients during the same period as for the IACI with general anesthesia and the MSA protocol. The ABR procedure was chosen for this comparison as it is performed in the same unit as the IACIs with the MSA protocol, involves the use of the same amount and type of resources, staff and time, and the medication costs are comparable. A hospital financial analyst provided and reviewed these calculations for accuracy.

After the MSA protocol was developed by the Sedation Service, with input from the Pediatric Rheumatology Division in late 2015, rheumatologists would identify patients in which IACI was clinically indicated and referred them to the Sedation Service for the MSA protocol. The Sedation Service would then reach out to the family to arrange the IACI procedure with the MSA protocol. The patients were advised that they were not required to fast prior to the procedure given that the MSA protocol is considered anxiolysis. Very young patients, those with contraindications to the administration of N_2_O, those requiring IACI of small joints, and patients whose parents preferred general anesthesia, were referred to the anesthesiology scheduler to have their procedure completed under general anesthesia.

### MSA protocol/ IACI procedure

Figure 1 illustrates the MSA protocol and IACI process. On the date of the IACI procedure with MSA protocol, upon arrival to the hospital, the patient and family registered and were escorted to a room in the Radiology Sedation Recovery Unit. The encounter started with the involvement of a certified child life specialist (CCLS) who met and greeted the patient and family as soon as they were placed in a room. The CCLS started working with the patient to build rapport and gain the child’s trust. The parents were allowed in the room and stayed next to the patient through the entire procedure. For communication with the patient, the CCLS was the “only voice in the room.”

A CCLS prepared the patient for each step of the process, providing appropriate choices and creating a coping plan to share with the team. The patient was desensitized to the N_2_O mask and the medication atomizer by providing a choice of mask flavor and allowing the patient to play with the mask and the atomizer. For distraction techniques, the patient was provided with choices such as: play a tablet video game, watch video on a tablet, listen to music or sing. The patient was also asked if he/she wished to observe the procedure, be informed of the steps of the procedure as they were happening, or none of the above. The CCLS reassessed the patient’s sensitivity, reaction, and cooperation throughout the procedure and if needed modified the distraction plan.

Medications as part of the MSA protocol were administered by a supervised advanced practice provider (APP) (nurse practitioner or a physician assistant) or a pediatric hospitalist. The practitioner administering the protocol medications was present in the room during the entire duration of the procedure. Thirty to forty minutes prior to N_2_O administration a topical numbing agent (lidocaine 4%) was applied to the skin over the injection site, ibuprofen 10 mg/kg, acetaminophen 15 mg/kg, and ondansetron orally disintegrating tablet (2 mg for children less than 20 kg and 4 mg for children greater than 20 kg) were administered. This was followed by IN fentanyl administration 10 to 15 min prior to N_2_O administration, at a dose of 2mcg/kg (maximal dose of 100 mcg). In addition, a vibrating device (Buzzy®) was placed on the proximal aspect of the extremity for as long as possible before the injection was performed, to help alleviate the pain sensation. N_2_O administration was started using a Belmed® mobile nitrous system. N_2_O was titrated up to a maximal concentration of 50% N2O/50% Oxygen, at a flow of 5 to 6 l/min. N_2_O was given for a minimal of 8 min before needle insertion.

The IACI was performed by a rheumatologist following nationally accepted practices, including aseptic preparation of the injection site and draping of the area with sterile towels. Next, a sterile 22 g needle attached to a syringe was inserted through the skin and subcutaneous tissues into the joint space using landmarks for appropriate placement. Aspiration of joint fluid was attempted with replacement of the filled syringe as needed without removal of the needle. With the needle still in place, a syringe with the appropriate amount of triamcinolone acetonide 40 mg/1 ml (dose 0.5 to 1 mg/kg per joint) was attached to the needle and the medication injected. Next, the syringe was again detached while the needle remained in the joint space and a syringe with 0.9% sterile sodium chloride solution was attached and injected to flush the needle and needle track. The needle was then withdrawn. The surgical site was bandaged.

Once the IACI was completed, N_2_O administration was discontinued and 100% oxygen was administered for five additional minutes. The sedationist performed a final patient assessment to make sure the patient had returned to baseline. The patient and family were then provided with post-procedure instructions and discharged home. Monitoring parameters included oxygen saturation and heart rate by pulse oximetry, as well as respiratory rate and mental status by direct observation.

## Results

A total of 80 patients underwent 121 joint injections. Forty-six patients had 1 joint injected, 28 patients had 2 joints injected, and 6 patients had 3 or 4 joints injected. Joints injected were predominantly knees and ankles (Table 1). Seventy-five percent (60/80) of the patients were females. Patient ages ranged from 2.5 to 18 years of age, with a median age of 13 IQR (9.5–15). Table 1 depicts the patient’s characteristics.

**Table 1:** Patient characteristics, joint features and pain scoring

Demographics (*n* = 80 patients)	Number (%)
Sex	
Female	60 (75)
Male	20 (25)
Age (years old)	
2–7	18 (22)
8–13	31 (39)
14–18	31 (39)
Joint Characteristics (*n* = 121 joints injected)	Number (%)
Number of joints injected	
1	46 (58)
2	28 (35)
3–4	6 (7)
Type of joint injected	
Knee	101 (86)
Ankle	16 (13)
Wrist	3 (2)
Elbow	1 (1)
Pain Scoring of procedure	Median (IQR)
Pain score (range 0–10)	
Patient (*n* = 72)^a^	1 (0–2.5)
Parent (*n* = 73)^a^	0 (0–2)
Rheumatologist (*n* = 78)^a^	0 (0–1)
Sedationist (*n* = 72)^a^	0 (0–1)

^a^There was no statistically significant difference between the pain ratings of different observers

### Procedure completion as planned

The procedure was completed as planned in 97.5% (78/80) of the patients. Two patients elected to undergo deep sedation due to increasing anxiety. These patients were excluded from the data analysis.

### Degree of motion during the procedure

Description of the amount of motion during the injection was available for 78 patients and was reported independently by the rheumatologist and the sedationist. The rheumatologist described the degree of motion as none in 68% (53/78) of the patients, mild in 35.5% (20/78) and moderate in 6.4% (5/78). The degree of motion as reported independently by the sedationist coincided with that of the rheumatologist. Severe motion was not observed in any patients (Table 2).

**Table 2:** Degree of motion, degree of pain and satisfaction with the procedure according to the patient, parent and providers

Scored outcomes	*n* (%)	*n* (%)	*n* (%)	*n* (%)
Degree of motion	None	Mild	Moderate	Severe
Rheumatologist (*n* = 78)	53 (68%)	20 (35.5%)	5 (6.4%)	0 (0%)
Sedationist (*n* = 78)	53 (68%)	20 (35.5%)	5 (6.4%)	0 (0%)
Degree of pain	None	Mild	Moderate	Severe
Pain score range	0	1–3	4–6	7–10
Patient (*n* = 72)	27 (37.5%)	33 (46%)	7 (9.5%)	5 (7%)
Parent (*n* = 73)	38 (52%)	23 (30%)	9 (13%)	3 (5%)
Rheumatologist (*n* = 78)	46 (59%)	23 (29.5%)	9(11.5%)	0 (0%)
Sedationist (*n* = 72)	49 (68%)	19 (26%)	4 (6%)	0 (0%)
Satisfaction with the procedure	Very satisfied	Satisfied	Somewhat satisfied	Not satisfied
Patient (*n* = 73)	67 (94.5%)	1 (1.4%)	3 (4.2%)	0 (0)
Parent (*n* = 76)	72 (97.4%)	1 (1.3%)	1 (1.3%)	0 (0)
Rheumatologist (*n* = 78)	73 (93.6%)	1 (1.3%)	4 (5.1%)	0 (0)
Sedationist (*n* = 77)	73 (94.8%)	1 (1.3%)	4 (5.2%)	0 (0)

### Degree of pain during the procedure

The degree of pain felt during the procedure was documented using a 0–10 verbal numeric scale with 0 being no pain and 10 being the maximal amount of pain possibly felt. Median pain scores were markedly low, with patient and parent median scores of 1 and 0, respectively, and providers (rheumatologists and sedationist), scoring a median of 0. Median pain scores and IQRs are described in Table 1.

#### Pain as reported by the patient/parent

Pain as reported by the patient was documented in 72 out of 78 patients. Of the 6 patients with missing data, pain was not documented in 3 charts and 3 patients were unable to comprehend the pain scale (ages ranging from 3 to 6 years old). All other patients were able to rate their pain with no difficulty. Using the categorization schema, the vast majority of the patients reported no pain (37%) or pain in the mild range (46%) as indicated in Table 2. Pain as reported by the parent was documented in 73 out of 78 patients. Half of the parents reported no pain, and the majority of the remaining reported pain in the mild range as indicated in Table 2.

#### Pain as reported by providers

Pain as reported by the rheumatologist and sedationist was documented in 78 patients. The majority of rheumatologists (59%) and sedationists (68%) reported interpretation of no pain and the majority of the remaining reported pain in the mild range as indicated in Table 2.

### Degree of satisfaction with the procedure

Satisfaction with the procedure reported by the patient, parent, rheumatologist and sedationist using a four-grade modified Likert scale (very satisfied, satisfied, somewhat satisfied, not satisfied) is shown in Table 2, with the vast majority (≥ 93% being very satisfied) and no one, neither patient, parent or provider, was dissatisfied.

A sub-analysis of pain scores and levels of satisfaction was performed between children that had 1 joint injection and children that had 2 or more joint injections, between children younger than 8 years of age and 9 years of age and older, and between children younger than 13 years of age and 14 years of age and older. No statistically significant difference was found between the subgroups included in these analyses (data not shown).

#### Adverse events

The only adverse event reported was emesis, which occurred in 2 patients (2.5%). No patient experienced changes in mental status or progression to a deeper level of sedation than intended. All patients remained interactive with the CCLS during the procedure.

#### Change in OR utilization during the study time period

Of the 133 patients that were referred to the Sedation and General Anesthesia services for their IAC during the frame time of the study, 80 patients (60%) had their ICAI performed by the sedation service and 53 (40%) were referred to general anesthesia in the OR due to parental preference, very young age (less than 2 years of age) with concern for inability to tolerate the N_2_O mask, or injection of small joints (fingers, temporo-mandibular joint). Prior to the implementation of the MSA protocol, 100% of the patients younger than 13 years of age and older patients who did not desire to have their IACI in the office with local anesthesia were referred to general anesthesia in the OR. This indicates a 60% decrease in utilization of the general anesthesia service for IACIs.

#### Cost savings per procedure

The mean cost of an IACI performed in the OR setting (under general anesthesia) was $1475 USD ± $744. The mean cost of a joint injection outside of the OR with the MSA protocol was estimated to be $948 ± $644 USD. This represents a 36% cost reduction.

## Discussion

We describe the use of a safe and effective MSA protocol to perform IACI in JIA patients. Although IACIs and the use of N_2_O/IN fentanyl to provide anxiolysis are well established procedures, there is no standard of care in treating pain during IACIs in the pediatric population and there is lack of literature describing how the procedure is done in children at most institutions. Some pediatric rheumatology units routinely use general anesthesia [4], as was the case in our institution prior to the establishment of the protocol described in this study. In addition, there are no studies on the effectiveness of the use of N_2_O for IACIs in the United States. This study is also valuable and unique as it provides an understanding of the cost effectiveness of performing the procedure outside of the OR avoiding the use of general anesthesia. The lower cost and more enjoyable and family-centered experience compared to general anesthesia in the OR are an example of how institutions can target certain procedures and patient populations to move towards more high-value care centered practices. The MSA protocol includes a combination of medications and distraction techniques that are well known, easy to administer and readily available at many pediatric institutions, making this protocol practical and simple to implement.

The combination of N_2_O/IN fentanyl along with a topical numbing agent, ibuprofen, acetaminophen, ondansetron, and CCLS used in our protocol allowed for a high procedure completion rate with adequate levels of pain control reflected in the none to mild levels of pain, low median pain scores and minimal motion manifested in the vast majority of the patients. Five and three patients felt severe pain according to the patient and to the parent respectively. The patients’ pain was transient and subsided right after the needle insertion. The patient’s attention was redirected by the CCLS and there was no requirement for additional doses of pain medications.

Two patients (ages 6 and 14) required deep sedation to complete the IACI procedure; this was anticipated in these patients from the time of their initial assessment due to severe anxiety, however the families chose to try the N_2_O/IN fentanyl protocol and to proceed to deep sedation only if needed.

We used a VNR scale to rate pain. The VNR scale is commonly used to measure pain intensity, especially in older children and adults [9]. When Tsze et al. evaluated the validity and reliability of the VNR scale for children experiencing acute pain they found the scale to have strong validity for most children aged 6 years and older, but not for children 5, 4 or younger [10]. A weakness of the present study is the lack of use of a scale for self-assessment of pain in children younger than 6 years of age. Seven of the patients in the present study were younger than 6 years of age. Despite this, only two of these patients (both of them 2 years old) were unable to understand the VNR to rate their pain and their pain rating was therefore not available. The other five children were 4 and 5 years old and had no difficulty understanding the VNR scale to rate their pain. Four of them rated their pain as 0 (and so did the parent, rheumatologist and sedationist) and 1 patient rated his pain as 3 (in the mild range). The parent, rheumatologist and sedationist independently rated this patient’s pain in the mild range as well: 2, 2 and 3 respectively.

Although we used a semi-quantitative measure to capture pain, described above, there are other factors that can attribute to perception of pain in our pediatric JIA population, that were not completely addressed in this study, such as, disease duration, pain at presentation, disease activity, functional disability and concurrent systemic immunomodulatory therapy; which can influence pain sensation in JIA patients [11, 12]. Identifying these factors might help develop a better pain management strategy for individual patients. As a small percentage of patients might still experience moderate/severe pain during IACIs, patients at risk for poor pain outcomes might be considered for deep sedation or general anesthesia for their procedure.

The combination of N_2_O/IN fentanyl along with a topical numbing agent, ibuprofen, acetaminophen, ondansetron, and CCLS used in the present study is intended for analgesia and anxiolysis (minimal sedation) and not for moderate or deep sedation. The administration of N_2_O and IN fentanyl are timed 20 min apart to allow for the Cmax (maximal concentration) of fentanyl to have passed [13–15] before the administration of N_2_O is initiated. This is in order to minimize the potential sedative and respiratory depressant effects of fentanyl [16] prior to the administration of 50% N_2_O (anxiolytic dose). Tracking of adverse events included special attention to changes in the patient’s level of consciousness. All the patients in the study remained alert and interacting with the CCLS during the procedure. These results are similar to those previously reported by Hoeffe et al. with the use of N_2_O /IN fentanyl for fracture reduction in the pediatric emergency room setting [17]. In their study the 57 patients that received the combination of 50% N_2_O/IN fentanyl remained minimally sedated (tired/sleepy with appropriate response to verbal conversation). This has also been the case with our unpublished experience with hundreds of patients receiving this medication combination for different minor painful procedures. As we consider this an anxiolysis protocol, it eliminates the need for patients to fast. It is important to note that N_2_O and fentanyl are emetogenic medications but as patients remain alert and their airway reflexes remain intact, the risk of emesis or regurgitation with subsequent pulmonary aspiration is minimized. Emesis occurred in 2 (2.5%) of the patients and no aspiration events occurred in our patient population. However, this study does not have the power or sample size required to detect aspiration related events. Our 2.5% incidence of emesis significantly less than the 11% incidence of emesis previously reported when using the combination of N_2_O and IN fentanyl in pediatric patients [17]. This is probably related to the addition of ondansetron to the MSA protocol.

In addition, with our MSA protocol, there is no requirement for the longer monitoring needed during the recovery phase after completion of the procedure under general anesthesia. This can shorten the amount of time families spend in the hospital for the procedure. Average time spent in our hospital for patients undergoing a short procedure like IACI in the OR is 3.2 h, with patients frequently spending 4 to 5 h in the hospital due to OR schedule delays or delays during the recovery phase. All the IACI non-OR MSA protocol encounters in our study had a duration of less than 2 h from the time the CCLS had the first interaction with the child, until the discharge of the patient. At the same time, the avoidance of general anesthetics allows for less risk of cardiovascular and respiratory complications [18].

The present study focuses on injection of large joints such as the knee and ankle. The protocol showed to be successful even when multiple large joints were injected. IACI of smaller joints was not evaluated in the present study as no patients required IACI of toes or fingers. The suitability of the MSA protocol for IACI of smaller joints needs to be evaluated, especially as these injections can be more difficult and more painful.

This is the first study to our knowledge that describes the involvement of a CCLS in addition to the use of N_2_O /IN fentanyl combined with other medicines to perform joint injections in children. The only other study that describes the use of distraction techniques used medical clowns [8]. CCLS use different patient interaction, distraction and desensitization techniques compared to those used by medical clowns. Having time for the CCLS to prepare the patient in a developmentally appropriate way and the use of distraction techniques are considered critical aspects for the success of N_2_O administration [19] and we cannot overemphasize the importance of these techniques and the presence of an experienced CCLS for the success of the MSA protocol.

Child selection also played a role in the success of the protocol. Although not specifically addressed in the study, older children are more distractible and more frequently able to tolerate the N_2_O mask on their face. At the same time, this might be responsible for some of the age distribution of our patient population, with only a small percentage (8.7%) being in the two to five-year-old range. Since very young children have difficulty tolerating the N_2_O mask on their face and remaining still, they tend to be scheduled for IACIs under general anesthesia. However, our study shows that even these young children can have a successful procedure experience with the use of our protocol, as no patient in this age range had to be converted to a full sedation due to pain, excessive motion, or lack of cooperation. Similarly, although in our experience most children older than 13 years of age can tolerate well the procedure in the office with the use of local or topical lidocaine only, some of these older children prefer additional pain and anxiety medication during the procedure. As demonstrated in the present study, these patients also do well with the MSA protocol.

Currently there are 4 acceptable methods to provide pain treatment during IACIs: general anesthesia, minimal sedation/anxiolysis, moderate and deep sedation, local anesthesia. As we have shown feasibility of an MSA protocol, future steps would entail a larger, multicenter study or survey comparing the different methods to provide further insight into the advantages and disadvantages of each technique. It would also be interesting for future studies to look at the levels of satisfaction with each of the methods.

One limitation of our study concerns our extrapolation of the total expenses to the hospital related to performing the IACI procedure by using costs associated with ABR testing for a comparable period. Future analyses will document costs directly related to observed IACI procedures performed with the MSA protocol, thereby permitting a more robust demonstration of the practice’s cost-effectiveness.

Our study is an example of how hospitals and clinicians can adopt high-value care practices while promoting patient and family-centered care outcomes. We believe the high levels of parent and patient satisfaction with the protocol are related in part to the families’ ability to be present during the procedure and patients’ ability to make choices. Although we did not officially survey all the patients who underwent joint injections with general anesthesia in the past, we received numerous comments from families and patients stating their preference for the procedure with the MSA protocol. The implementation of the MSA protocol for ICAI decreased general anesthesia use by the Division of Pediatric Rheumatology by 60%. This contributes to minimize resource utilization by eliminating the need to occupy a room in the OR and the additional personnel required to perform general anesthesia and monitor recovery of the patient afterwards. Overall the resource reductions contribute to the procedure being significantly less costly than general anesthesia when evaluated using activity-based cost accounting.

Currently, our institution is in the study year of a value-based payment model with a major payer whereby it will be paid a per member, per month capitation rate for Medicaid patients, thereby placing increased emphasis on identifying best clinical practices that minimize total cost of care and maximize value while improving patient and family experience.

## Conclusions

This study demonstrates that a MSA protocol including N2O and IN fentanyl in combination with a topical numbing agent, ibuprofen, acetaminophen, ondansetron, preparation and distraction techniques by a CCLS provides safe and effective analgesia for IACI in JIA patients while decreasing costs and delivering high-value, patient- and family-centered care.

## Abbreviations

ABR: Auditory brain stem response
APP: Advanced practice provider
CCLS: Certified Child Life Specialist
IACI: Intra-articular corticosteroid injection
IN: Intranasal
JIA: Juvenile Idiopathic Arthritis
MSA: Minimal Sedation/Anxiolysis
N_2_O: Nitrous Oxide
OR: Operating room
VNR: Verbal numeric rating
